# Evidence of Niche Partitioning under Ontogenetic Influences among Three Morphologically Similar Siluriformes in Small Subtropical Streams

**DOI:** 10.1371/journal.pone.0110999

**Published:** 2014-10-23

**Authors:** Karine Orlandi Bonato, Clarice Bernhardt Fialho

**Affiliations:** Universidade Federal do Rio Grande do Sul, Instituto de Biociências, Departamento de Zoologia, Programa de Pós-Graduação em Biologia Animal, CEP 91501–970, Porto Alegre, Rio Grande do Sul, Brazil; Onderstepoort Veterinary Institute, South Africa

## Abstract

Ontogenetic influences in patterns of niche breadth and feeding overlap were investigated in three species of Siluriformes (*Heptapterus* sp., *Rhamdia quelen* and *Trichomycterus poikilos*) aiming at understanding the species coexistence. Samplings were conducted bimonthly by electrofishing technique from June/2012 to June/2013 in ten streams of the northwestern state of Rio Grande do Sul, Brazil. The stomach contents of 1,948 individuals were analyzed by volumetric method, with 59 food items identified. In general *Heptapterus* sp. consumed a high proportion of *Aegla* sp., terrestrial plant remains and Megaloptera; *R. quelen* consumed fish, and Oligochaeta, followed by *Aegla* sp.; while the diet of *T. poikilos* was based on Simuliidae, Ephemeroptera and Trichoptera. Specie segregation was observed in the NMDS. Through PERMANOVA analysis feeding differences among species, and between a combination of species plus size classes were observed. IndVal showed which items were indicators of these differences. Niche breadth values were high for all species. The niche breadth values were low only for the larger size of *R. quelen* and *Heptapterus* sp. while *T. poikilos* values were more similar. Overall the species were a low feeding overlap values. The higher frequency of high feeding overlap was observed for interaction between *Heptapterus* sp. and *T. poikilos*. The null model confirmed the niche partitioning between the species. The higher frequency of high and intermediate feeding overlap values were reported to smaller size classes. The null model showed resource sharing between the species/size class. Therefore, overall species showed a resource partitioning because of the use of occasional items. However, these species share resources mainly in the early ontogenetic stages until the emphasized change of morphological characteristics leading to trophic niche expansion and the apparent segregation observed.

## Introduction

According to the competitive exclusion principle [1], species cannot coexist because competing for resources could lead to the exclusion of one or the other species or a population decrease. For coexistence to be possible, a niche differentiation would be required [2], [3].

This niche differentiation is known as resource partitioning that, according to [4], is any substantial difference in resource use between coexisting species. This resource partitioning would be the maintainer mechanisms of species biodiversity [5]. However, there is a neutral theory whose precept is that the diversity of species is the result of stochastic factors such as ecology drift, speciation, selection and dispersal acting at local and regional scale [6], [7]. Thus, in this theory is assumed that species have similar ecological needs and there is not a competitively superior species [6], [8]. Hubbell’s neutral model thus assumes that limited dispersal, rather than niche specialization, is the main explanation for spatial structure across ecological communities [9].

Other classic affirmation that tries to explain the involvement of interspecific competition in coevolution and complements the niche theory is “the ghost of competition past” [10]. Partitioning of resources can also be a consequence of competition past because in the past the species had a negative interaction and, during the evolutionary process, eventually developed distinct morphological and physiological characteristics that segregated it [2], [3]. Thereby, differences in trophic morphology, distinct habitat use, activity periods and tactical capture all minimize the effect of overlap [11], [12], [13].

The partitioning of resources may be influenced by factors such as time, space and ontogeny [4], [14], [15]. Therefore, these factors should be considered when we want to understand the mechanisms of fish species coexistence in streams [16]. Studies have demonstrated that the ontogenetic process may also be involved in resource partitioning by coexisting species [17], [18], [19]. Thus in order to segregation occur, differentiations in item consumption are necessary and these differences are related to size-dependent morphology, physiology and behavior [20], [21], [22]. The differences between sizes and stages of life are not restricted only to the features mentioned above, but also to energy requirement. This can lead individuals mainly the adults to use larger prey to maximize their energy intake [23].

Phylogenetically related species sharing morphological features especially in some stage of ontogenetic development. These related species tend to show ecological similarities [24] and, can be great instrument for studying the influence of ontogenetic factor in the coexistence of species. There is still a lack of studies that address a broader community context using three or more species [4], [19].

The Siluriformes are considered one of the most basal groups of fish and have 2,867 freshwater species and they have a diverse morphology, usually with benthic habits [25], [26]. Within the family Heptapteridae there are numerous genus including *Rhamdia* and *Heptapterus* [25], [26]. *Rhamdia quelen* is an opportunistic benthic species that can live in the midst of rocks, in deep wells, forages at night and near the margins searching for larger benthic macroinvertebrates and small fish [27], [28], [29], [30]. Species of the genus *Heptapterus* also live in crevices formed in rocky bottoms and in rapids, occupying low and medium depths, and are benthic [25]. The *Heptapterus* sp. used in this work is a new specie that is being described. *Trichomycterus poikilos* belongs to Trichomycteridae family and is a recently described species [31]. The species of *Trichomycterus* genus can feed during the day or at night revolving the substrate surface. They usually inhabit small water courses, strong currents and clear waters. Most of the species of the genus are *Trichomycterus* reported to live in streams with high circulation and bottom mainly composed of small stones and well oxygenated streams [32], [33], [34]. By having a relatively thin, elongated and depressed body they can explore the small spaces among rocks very well [35]. These three catfish have body elongate, trunk roughly cylindrical, head depressed, mouth wide and subterminal with small teeth distributed in three rows in the premaxilla and lower jaw (*T. poikilos*) or teeth in both jaws (*Heptapterus* sp.) or terminal mouth with small teeth inserted in dentigerous plates (*R. quelen*); they have maxilla and nasal barbels [32], [36], [37]. Therefore due to phylogenetic relationship, similar morphological and living habits are good tools for the study of coexistence of species.

Thus, this study was developed to test the hypothesis that three similar species of Siluriformes have a feed segregation influenced by ontogenetic process that allows the coexistence of species. Specifically, we tested the existence of dietary differences among three species and these differences are caused by interspecific variations related to the ontogenetic process by which individuals of these species spend. Therefore these variation combined with the use of non-limiting resources allows the coexistence of these species.

## Materials and Methods

### Ethics statement

Fish samples were colected with authorization n° 34940 from register n° 3196382 from Instituto Brasileiro do Meio Ambiente e dos Recursos Naturais Renováveis (IBAMA). IBAMA is the federal agency responsible for the environment in Brazil, and as such is responsible for emitting licenses to collect fish specimens according to Law N° 7,735 of February 22, 1989, in Brazil. This study was approved by Ethics Committee on Animal Use of the Universidade Federal do Rio Grande do Sul (Permit Number: 24434) and was conducted in accordance with protocols in their ethical and methodological aspects, for the use of fish. The committee follows National and International Norms and Guidelines, especially law 11.794 from November 8th, 2008 which disciplines the raising and use of animals for educational and research purposes. The study data presented herein are available as Table S1 and S2 files (e.g. stomach content analysis).

### Sampling

**Figure 1:**
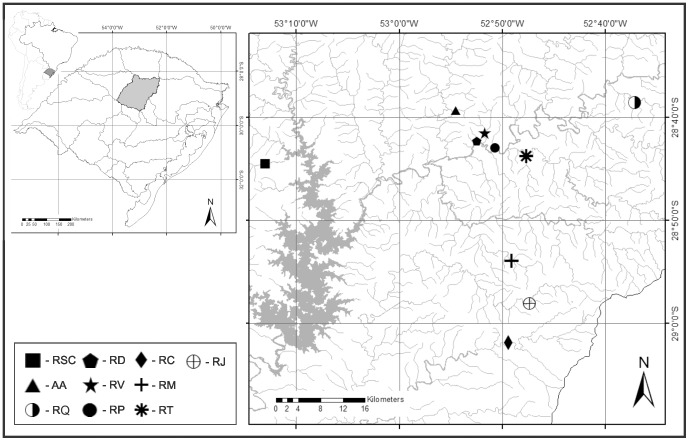
Sampling streams. Sampling streams in the Alto Jacuí sub-basin, state of Rio Grande do Sul, Brazil. For stream code see Material and Methods.

The study was conducted in ten streams (Figure 1) in northwest Rio Grande do Sul, which correspond to the Alto Jacuí sub-basin – Caixões River (RC) (S29° 01′ 54.4″/W 52° 49′ 25.1″); Jacuizinho River (RJ) (S 28° 58′ 02.9″/W 52° 47′ 20.3″); Morcego River (RM) (S 28° 53′ 55.0″/W 52° 49′ 05.6″); Turvo River (RT) (S 28° 43′ 47.0″/W 52° 47′ 40.4″), Valoroso Stream (RV) (S 28° 41′ 32.0″/W 52° 51′ 41.5″); Divinéia Stream (RD) (S 28° 42′ 16.7″/W 52° 52′ 25.9″); Arroio Angico (AA) (S 28° 39′ 17.9″/W 52° 54′ 31.1″); Paz Stream (RP) (S 28° 42′ 57.3″/W 52° 50′ 41.7″); Santa Clara River (RSC) (S 28° 44′ 30.1″/W 53° 13′ 03.0″); and Quati River (RQ) (S 28° 38′ 31.8″/W 52° 37′ 07.9″). All streams flow into the Jacuí River, which is one of the main tributaries to the Laguna dos Patos system and the streams are considered headwater streams.

Fish were collected in June, August, October and December 2012; February, April and June 2013. Each sampling event lasted four days. For the sampling, we used electrofishing with three stages of 30 min each, in stretches of 50 m per sampling stream. After sampling, fish were euthanized with 10% eugenol [38], [39], fixed in 10% formalin and then transferred to 70% alcohol for conservation. Fish were identified in the laboratory with identification keys and voucher specimens were deposited in the fish collection of the Departamento de Zoologia at the Universidade Federal do Rio Grande do Sul (*Rhamdia quelen* - UFRGS 19263, *Heptapterus* sp. - UFRGS 19266, *Trichomycterus poikilos* - UFRGS 19267). Individuals were dissected for stomach removal, which were then conserved in 70% alcohol.

### Diet Composition and Factors-Influence

Stomach contents were identified under optical and stereoscopic microscopes set to the lowest taxonomic level possible. Food items were identified using the identification keys [40], [41], [42] for invertebrates. They were then quantified according to the volumetric method (*i.e.*, the total volume of a food item consumed by the fish population given as a percentage of the total volume of all stomach contents [43]) using graduated test tubes and a glass counting plate [44].

The Nonmetric Multidimensional Scaling (NMDS) were used to verify the possible diet differentiation between the species. The NMDS consisting of an ordination technique that shows the distance between objects considered in accordance with a previously calculated dissimilarity matrix (Bray-Curtis) [45], [46]. After we used Permutational Multivariate Analysis of Variance (PERMANOVA) with 999 permutations [47] based on a dissimilarity Bray-Curtis matrix [46] to confirm statistically the existence of the difference between the species' diets and the influence of the factor specie plus size classes within each stream. This analysis was based on data volume. If a difference was found, the Indicator Value Index (IndVal) [48] was applied to get the food item indicators for each species and specie plus size classes also within each stream. The IndVal is based on a comparison of relative abundances and relative frequencies of the factors that are being tested in different groups selected *a priori* [49]. The greater specificity and fidelity of an item to a particular group, the greater the value of the indicator; and this method proves robust to differences within the group, sample sizes, and differences in abundance between the groups [50]. All analyzes were performed using R software [51] with the Vegan package [52].

### Trophic Niche Breadth and Feeding Overlap

Inferences about the level of specialization of overall species and they along their ontogenetic process were used the Levin’s measure [53] that was calculated for each species in each stream and to specie-class size in each stream using volume data. The Hurlbert’s formula [54] was applied to standardize the trophic niche measure (ranging from 0 to 1).

We used the Pianka's index [55] to estimate the species and size classes’ feeding overlap within each of the spatial-temporal units (seven samples months in each site). Overlap values range from zero to one, where zero indicates overlap absence and one indicates complete overlap. We established three categories to improve understanding of the overlap results, high overlap is given by overlap values >0.6, intermediate values overlap between 0.4–0.6 and low overlap values <0.4 [56]. Only groups represented by five or more individuals were used to comparisons or groups that contained similar numbers of individuals. To evaluate the significance of Pianka’s index [57] we used the null model with RA3 algorithm [58]. The null model performed 1,000 Monte Carlo randomizations for we can compare the created patterns (mean niche overlap values for all group pairs) with the those in the real data. In this model mean overlap values that are significantly lower than those expected by chance might indicate food partitioning, however values higher than those expected by chance might indicate food sharing [59]. Both the null model analysis and feeding overlap were computed using EcoSim 7.0 [57].

## Results

### Diet Composition and Factors-Influence

The contents of 1,984 stomachs (Table S1) belonging to three species (Table 1) were analyzed. We recorded 59 food items wherein *Heptapterus* sp. consumed a high proportion of *Aegla* sp. (34.7%), terrestrial plant remains (14.8%) and Megaloptera (12.8%) and *Rhamdia quelen* consumed fish (40.2%), and Oligochaeta (30.9%) followed by *Aegla* sp. (9.8%). The diet of *Trichomycterus poikilos* was based on Simuliidae (31.4%), Ephemeroptera (25.2%) and Trichoptera (18.7%) (Table S2).

**Table 1:** Taxonomic position of three species in the Alto Jacuí sub-basin.

Order/Family/Specie	Specie Code	Specie/Size Class Code	Size Classes (cm)
**SILURIFORMES**			
**Heptapteridae**			
*Heptapterus* sp.	H	H1	1 = 1.16–5.99;
		H2	2 = 6.00–10.99;
		H3	3 = 11.00–14.99;
		H4	4 = 15.00–19.74;
		H5	5 = 20.00–28.00
*Rhamdia quelen* (Quoy & Gaimard, 1824)	R	R1	1 = 1.42–4.99;
		R2	2 = 5.00–9.90;
		R3	3 = 10.00 15.90;
		R4	4 = 16.00–27.50
**Trichomycteridae**			
*Trichomycterus poikilos* Ferrer & Malabarba, 2013	T	T1	1 = 1.26–2.99;
		T2	2 = 3.00–4.99;
		T3	3 = 5.00–6.99;
		T4	4 = 7.00–8.93

Specie code of the three species studied, specie/size class code by each size class and size variation for each size class.

**Figure 2:**
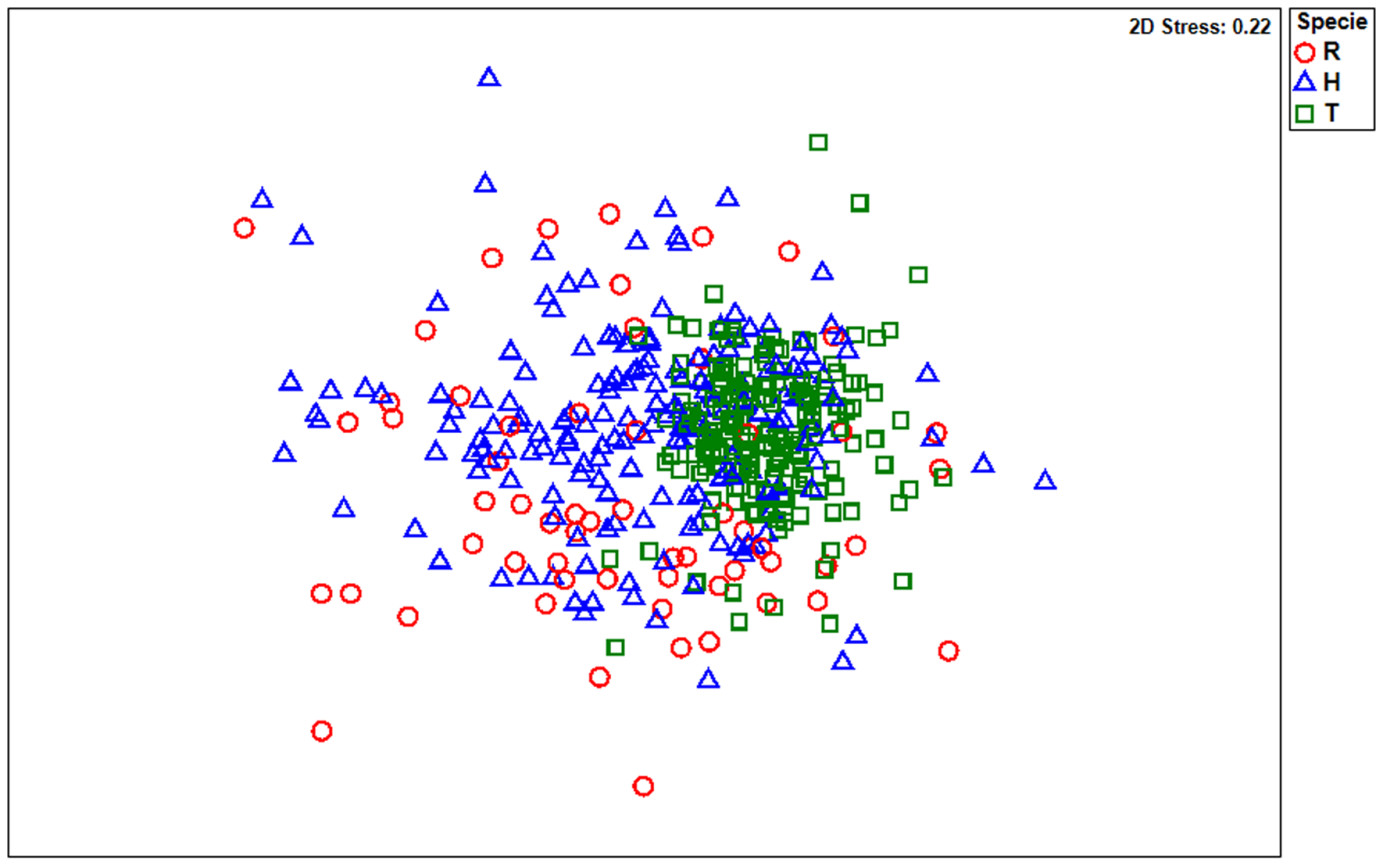
Two-dimensional plot of three fish species analyzed in Alto Jacuí sub-basin. The ordination resulting of the NMDS of the three species. For specie code see Table 1.

The NMDS analysis (Figure 2) showed the existence of a differentiation pattern among the species. In all streams the PERMANOVA analysis corroborated the resence of specie’s differentiation and the influence of factor class size (Table 2). The most significant food items that contributed to species and specie plus size class's differentiation were indicated by IndVal (Table 3, 4). The items indicated for IndVal for the species were the occasional items and in the major were indicated to *R. quelen* and *Heptapterus* sp. that have a more varied diet. The same occurred for the size class where the indicators items appeared most for larger classes by the use of occasional items.

**Table 2:** Permutational Multivariate Analysis of Variance results of each sampling stream in the Alto Jacuí sub-basin.

Stream	Specie Factor		Specie/Size Class Factor	
	F	*p*	F	*p*
**RC**	9.17	0.00	7.27	0.00
**RJ**	5.55	0.00	9.67	0.00
**RM**	8.33	0.00	3.00	0.01
**RV**	13.06	0.00	9.52	0.00
**RD**	16.19	0.00	9.36	0.00
**AA**	10.52	0.00	16.32	0.00
**RSC**	21.96	0.00	7.44	0.01
**RQ**	3.32	0.01	9.64	0.00
**RP**	21.78	0.00	13.34	0.00
**RT**	15.50	0.00	27.19	0.00

F and *p* values of specie and specie/size class factors. For stream code see Material and Methods.

**Table 3:** Indicator Value (IndVal), *p* value and Frequency of food items consumed by three species analyzed, discriminated among species.

Stream	Indicator Item	Specie	IndVal	*p*	Frequency
**RC**	Aquatic Lepidoptera larvae	R	0.704	0.05	2
	Bivalve	R	0.427	0.02	2
	Hymenoptera	R	0.380	0.02	2
	Ephemeroptera	H	0.679	0.03	193
**RJ**	Coleoptera larvae	R	0.645	0.00	10
	Adult Coleoptera	R	0.577	0.01	4
	Terrestrial insect remains	R	0.525	0.00	6
	Gastropoda	R	0.413	0.00	2
	Testae Amoebae	R	0.385	0.00	12
	Simuliidae	T	0.581	0.02	48
**RM**	Oligochaeta	R	0.816	0.00	8
	Coleoptera larvae	R	0.729	0.05	6
	Terrestrial plant remains	R	0.586	0.04	30
	Gastropoda	R	0.578	0.02	23
	Testae Amoebae	R	0.350	0.04	12
**RV**	Terrestrial plant remains	R	0.760	0.00	11
	Terrestrial insect remains	R	0.662	0.03	4
	Diptera Pupae	R	0.429	0.01	1
	*Aegla* sp.	H	0.974	0.01	7
	Megaloptera	H	0.964	0.02	3
**RD**	Oligochaeta	R	0.933	0.02	4
	*Aegla* sp.	R	0.918	0.00	12
	Terrestrial plant remains	R	0.855	0.00	24
	Odonata nymph	R	0.828	0.00	9
	Adult Coleoptera	R	0.822	0.00	5
	Terrestrial insect remains	R	0.765	0.03	13
	Trichoptera	R	0.727	0.00	67
	Animal organic matter	R	0.622	0.04	6
	Coleoptera larvae	R	0.595	0.00	8
	Hymenoptera	R	0.517	0.03	3
	Scale	R	0.504	0.03	5
	Simuliidae	H	0.701	0.00	85
	Chironomidae	H	0.575	0.00	92
**AA**	Oligochaeta	R	0.994	0.00	8
	Animal organic matter	R	0.800	0.02	2
	Aquatic insects remains	R	0.555	0.02	9
	Adult Coleoptera	H	0.511	0.05	4
	Simuliidae	T	0.711	0.02	106
**RSC**	Fish	R	0.999	0.02	1
	Oligochaeta	R	0.970	0.01	2
	Terrestrial Lepidoptera larvae	R	0.902	0.02	1
	Terrestrial insect remains	R	0.798	0.03	5
	Ephemeroptera	H	0.853	0.01	60
	Odonata nymph	H	0.813	0.04	3
	Sediment	H	0.380	0.03	11
**RQ**	Terrestrial insect remains	R	0.627	0.00	3
	Scale	R	0.518	0.00	7
	Adult Coleoptera	R	0.494	0.02	3
	Animal organic matter	R	0.481	0.02	2
	Terrestrial plant remains	R	0.408	0.04	14
	Gastropoda	R	0.390	0.00	5
**RP**	Fish	R	0.988	0.04	4
	Terrestrial insect remains	R	0.474	0.00	5
**RT**	Nematoide	R	0.795	0.03	9
	Orthoptera	R	0.713	0.04	3
	Scale	R	0.650	0.03	9
	Coleoptera larvae	R	0.638	0.04	6
	Adult Coleoptera	R	0.623	0.04	4
	Diplopoda	R	0.476	0.03	1
	Terrestrial Hemiptera	R	0.462	0.04	2
	Detritus	R	0.339	0.04	1
	Megaloptera	H	0.921	0.04	6
	*Aegla* sp.	H	0.895	0.04	26
	Odonata nymph	H	0.509	0.04	8

Only items with significant values *p*<0.05 are listed. For stream and specie code see Material and Methods and Table 1.

**Table 4:** Indicator Value (IndVal), *p* valeu and Frequency of food items consumed by three species analyzed, discriminated among species/size classes.

Stream	Indicator Item	Specie/Size Class	IndVal	*p*	Frequency
**RC**	Bivalve	R3	0.249	0.01	2
	Hymenoptera	R3	0.174	0.01	2
	Fish	H1	0.907	0.03	6
	Megaloptera	H1	0.848	0.02	9
	Aquatic insects remains	H4	0.738	0.01	12
	Aquatic Hemiptera	H4	0.381	0.05	3
	Scale	H4	0.156	0.02	6
**RJ**	Adult Coleoptera	R3	0.438	0.01	4
	*Aegla* sp.	H4	0.949	0.02	12
	Fish	H4	0.836	0.03	1
	Scale	H5	0.542	0.01	4
	Rvegsu	H5	0.438	0.02	17
	Simuliidae	T4	0.704	0.01	48
**RM**	Terrestrial plant remains	R3	0.493	0.01	30
	Adult Coleoptera	R3	0.490	0.02	6
	Gastropoda	R3	0.376	0.04	23
	Animal organic matter	H3	0.732	0.01	10
	Rvega	H3	0.164	0.02	6
**RV**	Diptera Pupae	R4	0.143	0.03	1
	Fish	H3	0.815	0.04	1
	Adult Coleoptera	H3	0.143	0.04	1
	Ephemeroptera	T4	0.585	0.03	70
**RD**	Terrestrial plant remains	H4	0.787	0.01	24
	Aquatic insects remains	H4	0.654	0.04	12
	Coleoptera larvae	H4	0.308	0.05	8
	Simuliidae	T4	0.469	0.00	85
**AA**	Oligochaeta	R2	0.985	0.03	8
	Animal organic matter	R2	0.584	0.03	2
	Aquatic insects remains	R2	0.283	0.03	10
	Megaloptera	H3	0.938	0.04	4
	*Aegla* sp.	H5	0.984	0.01	4
	Rit	H5	0.349	0.03	3
**RSC**	Fish	R4	0.997	0.02	1
	Oligochaeta	R4	0.942	0.01	2
	Terrestrial Lepidoptera larvae	R4	0.818	0.02	1
	Terrestrial insect remains	R4	0.656	0.00	5
	Ephemeroptera	H3	0.649	0.05	61
	Sediment	H3	0.148	0.04	11
	Simuliidae	T4	0.641	0.04	64
**RQ**	Scale	R2	0.289	0.05	7
	Terrestrial insect remains	R3	0.445	0.04	3
	*Aegla* sp.	H3	0.739	0.04	11
	Odonata nymph	H4	0.249	0.01	2
**RP**	Fish	R4	0.990	0.02	5
	Nematoide	R4	0.375	0.04	4
	Chironomidae	T4	0.222	0.02	70
**RT**	Detritus	R1	0.106	0.00	1
	Scale	R2	0.301	0.05	9
	Coleoptera larvae	R2	0.296	0.04	6
	Diplopoda	R2	0.162	0.05	1
	Terrestrial Hemiptera	R2	0.153	0.04	2
	Nematoide	R3	0.776	0.01	9
	Orthoptera	R3	0.654	0.02	3
	Sediment	H5	0.488	0.04	47

Only items with significant values *p*<0.05 are listed. For stream and specie/size class code see Material and Methods and Table 1.

### Trophic Niche Breadth and Feeding Overlap

**Figure 3:**
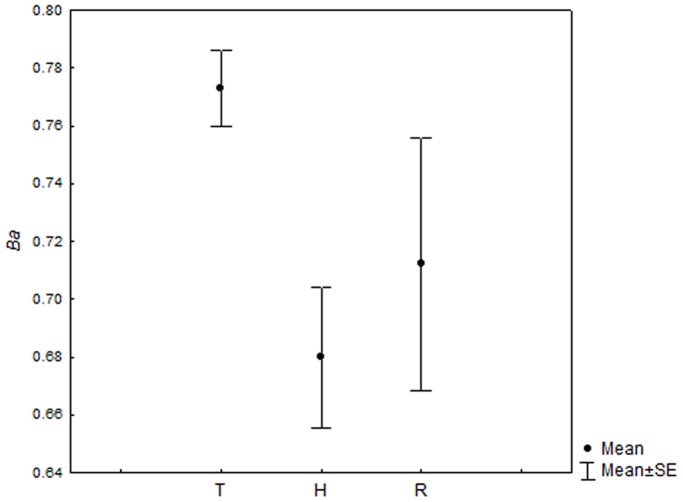
Values of trophic niche breadth for fish species analyzed in Alto Jacuí sub-basin. Values of trophic with mean±standart error for each specie analyzed in sampling units. For specie code see Table 1.

**Figure 4:**
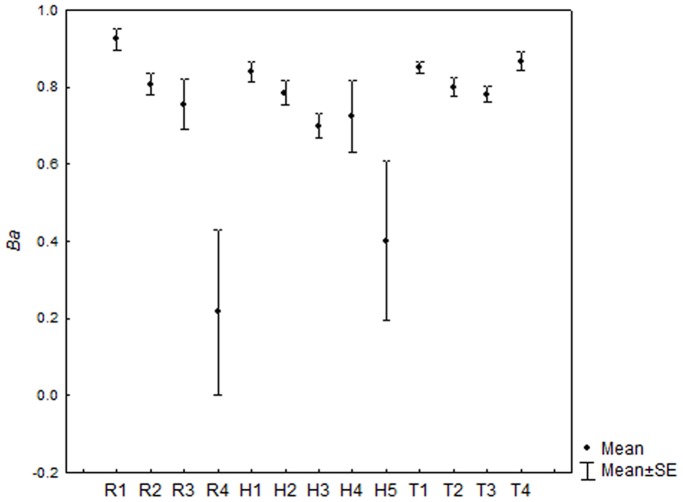
Values of trophic niche breadth for size class of each fish specie analyzed in Alto Jacuí sub-basin. Values of trophic with mean±standart error for each specie/size class analyzed in sampling units. For specie/size class code see Table 1.

Niche breadth values were high (>0.61) for all species in all streams. The mean values were higher for *T. poikilos* (*Ba*: 0.777) and *R. quelen* (*Ba*: 0.727) than *Heptapterus* sp. (*Ba*: 0.693) (Figure 3). Overall for size classes 92.7% of niche breadth values were high and the few intermediate (0.4–0.61) as R4, H4 and H5. There is an abrupt niche breadth reduction in the larger size classes for *R. quelen* and *Heptapterus* sp. However *T. poikilos* maintain a more uniform niche breadth (Figure 4).

**Figure 5:**
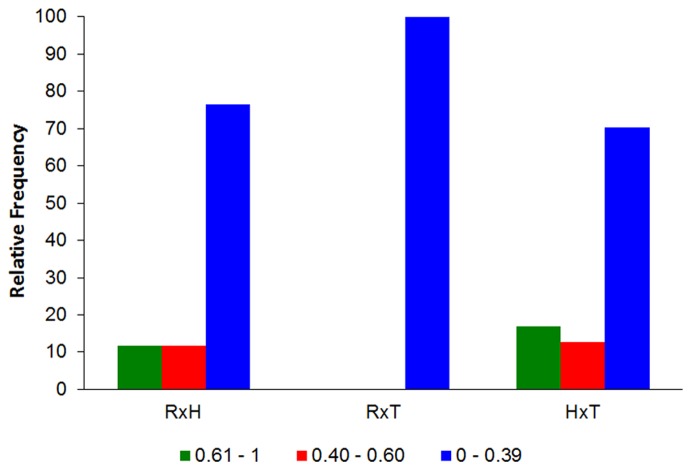
Pianka's index results. Relative frequency of Pianka's index of all pairwise interactions between the species analyzed per sampling unit. For specie code see Table 1.

Feeding overlap values for species were mostly low (0−0.4) in all spatial-temporal units. *Rhamdia quelen* and *T. poikilos* only had low feeding overlap values. However the interaction between *R. quelen* with *Heptapterus* sp. showed larger quantitative of low overlap, and about 12% were of high and intermediate values. The largest number of high feeding overlap were the interaction between *Heptapterus* sp. and *T. poikilos* (Figure 5). Most of observed values (84%) were not significantly higher than those expected by chance. This result indicates a resource partitioning among the species.

**Figure 6:**
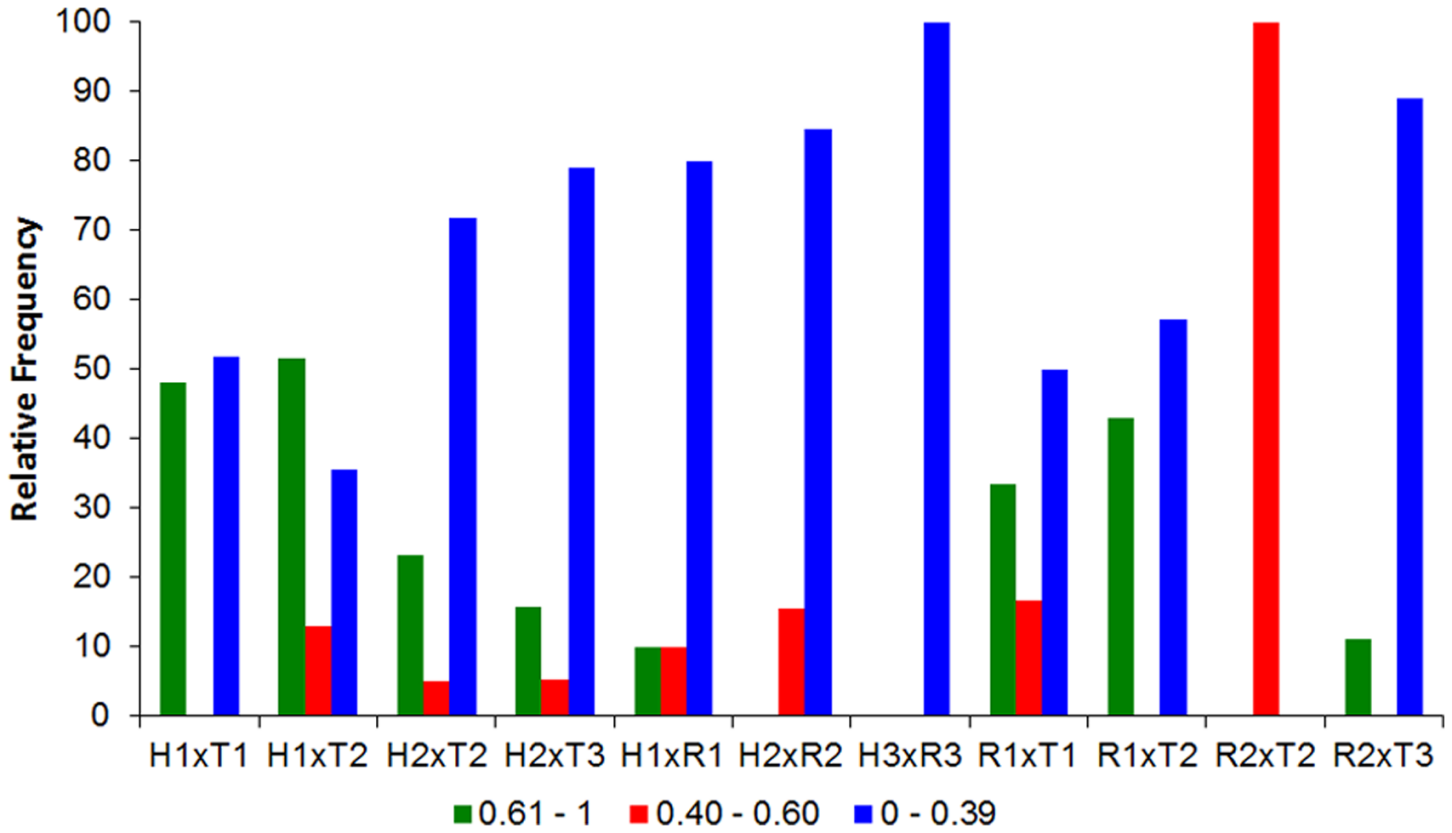
Pianka's index results. Relative frequency of Pianka's index of all pairwise interactions between the species with compatible size classes per sampling unit. For specie/size class code see Table 1.

**Figure 7:**
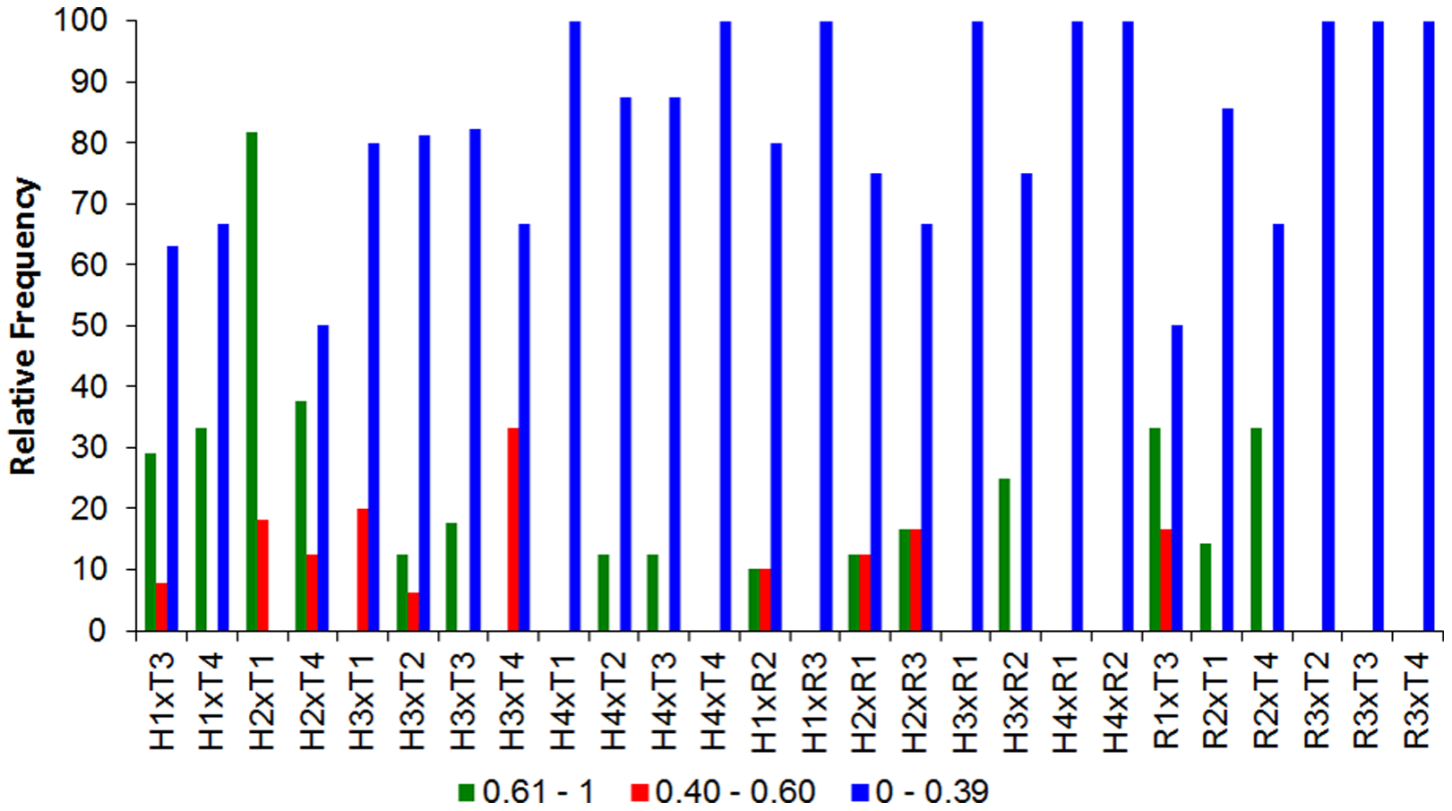
Pianka's index results. Relative frequency of Pianka's index of all pairwise interactions between the species with not compatible size classes per sampling unit. For specie/size class code see Table 1.

Investigating the feeding overlap values for species and their class size we observed that the most of feeding overlaps remains were low. However high and intermediate feeding overlaps were in largest frequency only in smaller size classes independently of these size are equivalents (Figure 6, 7). Observed values were significantly higher than those expected by chance in 60% of the feeding overlap interactions, showing the major of the spatial-temporal units for size class is occurring resource sharing.

## Discussion

The partitioning resource found for the three species of Siluriformes in all streams sampled is relates with the differential use of resources by the species. Food items that were responsible for these differences varied from a stream to another, but overall the items were used in greater quantity or frequency by species. The indicator items for *R. quelen* and *Heptapterus* sp. were of the occasional use, higher frequency and larger size. The indicator item for *T. poikilos* appeared rarely and when appeared was Simuliidae larva that was widely used.

The high consumption of fish, Oligochaeta and *Aegla* sp. by *R. quelen* indicate a carnivorous/piscivorous habit. This habit is described in the literature [27], [60], [61]. *Heptapterus* sp., which was considered an invertivorous species, ingested large amount of *Aegla* sp., terrestrial plant remains, and Megaloptera- though in smaller proportions (but with higher frequency) fed on aquatic insects such as Ephemeroptera and Trichoptera. *Heptapterus* sp. was also reported as invertivorous but as ingesting a much larger amount aquatic larvae invertebrates [60] and two species of *Heptapterus* are classified within the guild of those that mainly eat items from their aquatic environment [62]. *Trichomycterus poikilos* was shown to be insectivorous, eating mostly aquatic larvae of Simuliidae, Ephemeroptera and Trichoptera. This insectivorous diet with ingestion of benthic larvae is well reported in the literature for other species of the genus *Trichomycterus* [63], [64], [65], [66], [67].

The diet differentiation were observed in the ontogenetic level when we analyzed the specie plus size class factor. In this case over again the indicator items were those used occasionally by the larger size classes. The influence of this factor has been studied in Neotropical streams and is connected to the innumerous features that change with the fishes development, be they morphological and physiological characteristics (e.g. increasing individual size, mouth gap, changes in the digestive tract) or even behavioral habits (e.g. locomotion ability) [68], [69], [70]. The more features that shift over development and that differentiate these species are related to the size that can achieve *R. quelen* and *Heptapterus* sp. The expressive growth these species present during development allows a larger mouth gape can allowing the use of larger items like *Aegla* sp., fish, Megaloptera, Gastropoda, terrestrial insect remains and Oligochaeta by the larger sizes. In this study the difference between the largest and smallest individuals was 26.84 cm and 26.08 cm for *Heptapterus* sp. and *R. quelen* respectively. With the increase in fish size the individuals need to maximize their energetic gain by ingesting larger prey with a higher caloric [71]. Sometimes the ontogenetic diet shifts may be seen as a consequence of the absolute size increment of their mouth gape [72], the larger fish size enables ingestion of larger preys items due to an increase in searching ability and capture efficiency [73], [74].

*Trichomycterus poikilos* does not achieve a largest size than other species and by having a relatively thin, elongated and depressed body they can explore places not explore by the other species [35] as a explore the small spaces among rocks very well, what seems to have occurred in the present study. So, the items used by *T. poikilos* were smaller size however the ingestion of fish, *Aegla* sp. and terrestrial plant remains occurred only in the larger length classes. Ontogenetic differences in the diet of two *Trichomycterus* species (*T. crassicaudatus* and *T. stawiarski*) were also detected by [67]. *Trichomycterus chiltoni* showed intraspecific differences in its diet which were related to ontogeny because the species reached a larger size which allowed for the consumption of larger prey (therefore broadening the trophic spectrum because of morphological characteristics such as the mouth and body size) [75]. This differentiation of diet by influence of ontogeny was also found by many others studies [70], [76], [77], [78], [79].

Dietary differences cited above are also supported by the niche breadth data. All species showed high niche breadth. Most of items were ingested occasionally, expanding the trophic spectrum of these species that have a diverse diet and wide niche breadth. This broad food spectrum is already expected in Neotropical stream fish, because such streams have a wide range of available resources [27], [61], [80], [81], [82]. Neotropical stream fish have a tendency towards generalism thus having the ability of trophic plasticity [83].

The mean niche breadth values were low only in the high size class of *R. quelen* and *Heptapterus* sp. In the early stages of life the species tend to exhibit more generalist behavior and with increasing body size they begin to exhibit more specialist behavior, as was found by [19] when they studied ontogenetic diet shifts among five species of *Crenicichla*.

The low overlap among the three species also indicates the resource partitioning. There are studies that show *R. quelen* and *Heptapterus* sp. not overlapping [61] and *R. quelen* overlapped with *Trichomycterus* sp. [27]. However, we believe that much of this low overlap was perceived because we did not group food items into broad categories- a discussion approached by other authors [27], [66], [84]. This in our opinion becomes very important in this type of analysis where one can notice the feeding preference of the species for certain aquatic larvae, for example, the strong preference of *T. poikilos* for Simuliidae larvae. If we think in broader trophic categories, we definitely would have an increased dietary overlap of these species. In overall the overlap niche values were low, however only in smaller size classes had high values. The null model showed that there is a resource sharing between the species/size class, this pattern were seen in all spatial-temporal units. The items that are shared by species are those that are most abundant (personal observations) and it is known that the abundance of some items is often responsible for the coexistence of species [77]. Sharing of resources does not mean existence of competition; the high abundance of resources and stochastic processes can promote relaxation of interspecific competition and facilitating coexistence which was also reported in other study [85]. We note that most items found in this study are shared by species, with overlap avoided both by the abundance of items in the aquatic system [4], [27], [54] and by the different microhabitats used for feeding, periods of activity, and tactics of capture. This pattern of features that avoid overlap and often explain the coexistence of sympatric species is well reported in Neotropical streams (e.g. [28], [86], [87], [88], [89], [90]).

It is very difficult and complex we make inferences about species coexistence [85], [91]. The three Siluriformes species studied here are coexisting, but the force that allows this coexistence is difficult to prove. The current scenario shows species with morphological similarities, partitioning some resources and sharing abundant resources resulting no competition among them (4). But we cannot say with absolute certainty that the current scenario has not been structured over time through large negative pressures of the past between these species [10], [92]. Differences in the species population numbers in different sample replicates (Table S1) was seen, and this may be related to stochastic events and other precepts of the neutral theory or which competition became more abundant a specie at a stream than other [93]. However there is no evidence that the closest similarities in these streams had higher population numbers. To prove this theory we must increase our knowledge of dispersal of fish in continental basins, because we know of dispersal limitation in aquatic environments [94] and their phylogenetic relationships [95].

In conclusion, our hypothesis was accepted, there is food segregation of three species of Siluriformes studied and it is related to the differential use of items in different stages of life. This because the differences in diet among species are related to morphological differences and life habits. There are more pronounced ontogenetic changes in *Heptapterus* sp. and *R. quelen* than in *T. poikilos*, given mainly by shifts in the morphology of these species. This process of diet shift throughout species growth is undoubtedly a way to expand or shift the trophic niche of the species in order to avoid inter- and intraspecific competition and maintain species coexistence that also maintain for the sharing resource of abundant items.

## Supporting Information

Table S1 **Number of analyzed stomachs per sample unit (stream and sampled month) for each size class for the species studied.** For stream and specie/size class code see Material and Methods and Table 1.
(DOCX)

Table S2 **Stomach content analyzed (% by volume) for the species of Siluriformes sampling in ten streams in Alto Jacuí sub-basin.** For species and streams code see Material and Methods and Table 1. Asterisk indicates values less than 0.1%.
(DOCX)
