# Supplementary material for: Evidence of Niche Partitioning under Ontogenetic Influences among Three Morphologically Similar Siluriformes in Small Subtropical Streams
Source: PLoS One. 2014 Oct 23;9(10):e110999. doi: 10.1371/journal.pone.0110999 (PMC4207772; doi:10.1371/journal.pone.0110999)
Supplement: Table S1 — Number of analyzed stomachs per sample unit (stream and sampled month) for each size class for the species studied. For stream and specie/size class code see Material and Methods and Table 1. (DOCX) [file pone.0110999.s001.docx]

**Table S1.**

| **Stream** | **Sampled Month** | **R1** | **R2** | **R3** | **R4** | **H1** | **H2** | **H3** | **H4** | **H5** | **T1** | **T2** | **T3** | **T4** |
| --- | --- | --- | --- | --- | --- | --- | --- | --- | --- | --- | --- | --- | --- | --- |
| **RC** | Jun/12 | - | - | - | - | 13 | 10 | 1 | - | - | 1 | 1 | - | - |
|  | Ago/12 | - | - | - | - | 6 | 18 | 3 | 1 | - | - | 5 | 3 | - |
|  | Out/12 | - | - | - | - | 4 | 10 | - | - | - | - | 4 | - | - |
|  | Dez/12 | - | - | - | - | 9 | 17 | 2 | - | - | 1 | 1 | 4 | - |
|  | Fev/13 | - | 1 | 1 | - | 12 | 10 | 7 | 1 | - | 5 | 10 | 6 | - |
|  | Abr/13 | - | - | - | - | 18 | 8 | 2 | - | - | 6 | 17 | 7 | - |
|  | Jun/13 | 1 | 1 | - | - | 10 | 14 | 5 | 1 | - | - | 20 | 5 | - |
| **RJ** | Jun/12 | - | 2 | 1 | - | 6 | 3 | 1 | - | - | - | 3 | 9 | - |
|  | Ago/12 | - | 1 | 1 | - | 6 | 4 | 2 | - | - | - | - | 4 | - |
|  | Out/12 | - | - | - | - | - | - | - | - | - | - | - | - | - |
|  | Dez/12 | - | - | - | - | 7 | 1 | 1 | - | - | 3 | 2 | 6 | - |
|  | Fev/13 | 3 | 2 | 1 | - | 2 | 7 | - | 1 | - | 9 | 7 | 5 | - |
|  | Abr/13 | 6 | 2 | 2 | - | 6 | 9 | 1 | - | 1 | 12 | 13 | 5 | - |
|  | Jun/13 | 1 | - | - | - | 10 | 4 | - | 1 | - | 13 | 6 | 7 | 1 |
| **RM** | Jun/12 | 2 | 4 | 3 | - | 10 | 10 | - | 1 | - | - | 1 | - | - |
|  | Ago/12 | - | 3 | 1 | - | 8 | 6 | - | - | - | - | - | - | - |
|  | Out/12 | - | 1 | 1 | - | 5 | 5 | 2 | - | - | - | - | - | - |
|  | Dez/12 | 2 | 2 | 1 | - | 7 | 2 | - | - | - | - | - | - | - |
|  | Fev/13 | 4 | 1 | - | - | 21 | 3 | 1 | - | - | - | 5 | - | - |
|  | Abr/13 | 3 | - | 3 | - | 16 | 5 | 1 | - | - | - | - | - | - |
|  | Jun/13 | 2 | 4 | 1 | - | 15 | 8 | 4 | - | - | - | 3 | - | - |
| **RV** | Jun/12 | - | - | - | 1 | - | - | - | - | - | - | - | 5 | - |
|  | Ago/12 | - | - | - | - | - | - | - | - | - | - | - | 8 | - |
|  | Out/12 | - | - | - | - | - | - | - | - | - | - | 2 | 5 | - |
|  | Dez/12 | - | - | - | - | - | - | - | - | - | - | - | - | - |
|  | Fev/13 | - | - | - | - | 4 | 2 | - | 1 | 1 | 15 | 7 | 4 | - |
|  | Abr/13 | - | - | - | - | 2 | - | - | - | - | 14 | 9 | 4 | - |
|  | Jun/13 | - | - | - | - | 4 | 2 | - | - | - | 4 | 21 | 4 | 1 |
| **RD** | Jun/12 | - | - | - | - | - | - | - | - | - | - | - | - | - |
|  | Ago/12 | - | - | - | - | 2 | 1 | - | 1 | - | 1 | 2 | 5 | - |
|  | Out/12 | - | - | - | - | 4 | 9 | 3 | 2 | - | - | 19 | 10 | - |
|  | Dez/12 | - | - | - | - | 5 | 9 | 2 | 2 | - | 2 | 6 | 15 | - |
|  | Fev/13 | - | - | - | - | 1 | 4 | 2 | 1 | - | 2 | 4 | 13 | - |
|  | Abr/13 | - | - | - | - | 3 | 5 | 2 | 2 | - | 3 | 5 | 16 | 1 |
|  | Jun/13 | - | - | - | - | 3 | 3 | 2 | 2 | - | 2 | 11 | 12 | 2 |
| **AA** | Jun/12 | - | - | - | - | - | 1 | - | - | - | - | 2 | 2 | - |
|  | Ago/12 | - | - | - | - | 1 | - | - | - | - | - | 2 | 2 | - |
|  | Out/12 | - | 1 | - | - | 2 | 5 | 1 | - | - | 1 | 4 | 23 | - |
|  | Dez/12 | 2 | - | - | - | 12 | 3 | 1 | - | - | 8 | 6 | 15 | - |
|  | Fev/13 | - | - | - | - | 7 | 1 | 1 | - | 1 | 1 | 11 | 9 | - |
|  | Abr/13 | - | - | - | - | 7 | 1 | 2 | - | - | 7 | 9 | 13 | - |
|  | Jun/13 | - | 2 | - | - | 4 | 2 | - | - | - | 3 | 16 | 7 | 2 |
| **RSC** | Jun/12 | - | - | - | - | - | 2 | 2 | - | - | 1 | 8 | 6 | 1 |
|  | Ago/12 | - | - | - | - | - | 1 | 2 | - | - | - | 4 | 2 | - |
|  | Out/12 | - | - | - | - | - | 1 | - | - | - | - | 1 | 1 | 1 |
|  | Dez/12 | - | - | - | 1 | - | - | - | - | - | 1 | 1 | 1 | 1 |
|  | Fev/13 | - | - | - | - | - | - | - | - | - | 1 | 1 | 1 | 1 |
|  | Abr/13 | - | - | 1 | - | 1 | - | - | - | - | 1 | 1 | 1 | 1 |
|  | Jun/13 | - | - | - | - | - | - | - | - | - | 1 | 1 | 1 | 1 |
| **RQ** | Jun/12 | - | - | - | - | 2 | 2 | 1 | - | - | - | 2 | 3 | - |
|  | Ago/12 | - | - | - | - | 1 | 4 | - | - | - | - | 3 | 15 | - |
|  | Out/12 | - | 2 | - | - | 1 | 3 | 2 | - | - | - | 4 | 5 | - |
|  | Dez/12 | 2 | 1 | 1 | - | 3 | 4 | - | - | - | 11 | 2 | 2 | - |
|  | Fev/13 | 4 | 1 | 1 | - | 6 | 2 | 1 | 1 | - | 8 | 3 | - | - |
|  | Abr/13 | 4 | 5 | - | - | 6 | 2 | - | - | - | 8 | 5 | 3 | - |
|  | Jun/13 | - | - | - | - | 7 | 1 | 1 | - | - | 8 | 14 | 4 | - |
| **RP** | Jun/12 | - | 1 | - | - | - | 1 | - | - | - | - | 1 | 1 | - |
|  | Ago/12 | - | - | - | - | 3 | 2 | 2 | - | - | - | 7 | 8 | 1 |
|  | Out/12 | - | 1 | - | 1 | 2 | 1 | 2 | - | - | - | 10 | 5 | - |
|  | Dez/12 | - | 1 | - | 1 | 4 | 2 | - | 1 | - | 4 | 2 | 5 | - |
|  | Fev/13 | - | - | - | - | 4 | - | - | 1 | - | 6 | 4 | 10 | - |
|  | Abr/13 | - | 2 | - | - | - | 1 | - | 2 | - | 3 | 5 | 5 | 1 |
|  | Jun/13 | - | 1 | 2 | - | 1 | 2 | 1 | 1 | - | - | 7 | 6 | - |
| **RT** | Jun/12 | - | - | - | - | 4 | 6 | 5 | 4 | 1 | - | 15 | 5 | 7 |
|  | Ago/12 | - | - | - | - | 11 | 12 | 1 | 4 | 1 | - | 14 | 6 | 6 |
|  | Out/12 | - | 1 | 1 | - | 5 | 15 | 1 | 1 | - | - | 10 | 10 | 5 |
|  | Dez/12 | 1 | 3 | - | - | 4 | 4 | 4 | 1 | 2 | 9 | - | 11 | 4 |
|  | Fev/13 | - | 2 | - | - | 7 | 6 | 2 | 1 | - | 5 | 5 | 5 | 3 |
|  | Abr/13 | - | 1 | 1 | - | 9 | 11 | 7 | 2 | 1 | 4 | 9 | - | 8 |
|  | Jun/13 | - | 1 | - | - | 14 | 5 | 2 | 3 | - | 1 | 14 | 6 | 5 |
