# Supplementary material for: Evidence of Niche Partitioning under Ontogenetic Influences among Three Morphologically Similar Siluriformes in Small Subtropical Streams
Source: PLoS One. 2014 Oct 23;9(10):e110999. doi: 10.1371/journal.pone.0110999 (PMC4207772; doi:10.1371/journal.pone.0110999)
Supplement: Table S2 — Stomach content analyzed (% by volume) for the species of Siluriformes sampling in ten streams in Alto Jacuí sub-basin. For species and streams code see Material and Methods and Table 1. Asterisk indicates values less than 0.1%. (DOCX) [file pone.0110999.s002.docx]

**Table S2.**

| **Food Items** | **RC** |  |  | **RJ** |  |  | **RM** |  |  | **RV** |  |  | **RD** |  | **AA** |  |  | **RSC** |  |  | **RQ** |  |  | **RP** |  |  | **RT** |  |  |
| --- | --- | --- | --- | --- | --- | --- | --- | --- | --- | --- | --- | --- | --- | --- | --- | --- | --- | --- | --- | --- | --- | --- | --- | --- | --- | --- | --- | --- | --- |
|  | **R** | **H** | **T** | **R** | **H** | **T** | **R** | **H** | **T** | **R** | **H** | **T** | **H** | **T** | **R** | **H** | **T** | **R** | **H** | **T** | **R** | **H** | **T** | **R** | **H** | **T** | **R** | **H** | **T** |
| **Calliphoridae** | 0 | 0 | 0 | 0 | 0 | 0 | 0 | 0 | 0 | 0 | 0 | 0 | 0 | 0 | 0 | 0 | 0 | 0 | 0 | 0 | 0 | 0 | 11 | 0 | 0 | 0 | 0 | 0 | 0 |
| **Ceratopogonidae** | 0 | 0 | 0 | 0.1 | * | 0.1 | * | 0 | 0 | 0 | 0 | 0 | 0 | 0 | 0 | 0 | * | 0 | 0 | 0 | 0 | 0 | 0.3 | 0 | 0 | 0 | 0 | 0 | * |
| **Chironomidae** | 0.3 | 0.2 | 1.6 | 0.1 | 0.3 | 6 | * | 0.6 | 3 | 0 | 0.1 | 4.2 | 0.2 | 11.4 | * | 0.2 | 3.3 | 0 | 1 | 5.3 | 1.9 | 1.4 | 9 | 3.6 | 0.1 | 14.6 | * | 0.5 | 13.6 |
| **Culicidae** | 0 | 0 | 0 | 0 | 0 | 0 | 0 | 0 | 0 | 0 | 0 | 0 | 0 | 0 | 0 | 0 | 0 | 0 | 0 | 0 | 0 | 0 | 0 | 0 | 0 | 0 | 0 | * | * |
| **Empididae** | 0 | 0 | 0 | 0 | 0 | 0 | 0 | 0 | 0 | 0 | 0 | 0 | 0 | 0.2 | 0 | 0 | * | 0 | 0 | 0.2 | 0 | 0 | 0 | 0 | 0 | 0 | 0 | * | 0.1 |
| **Psycodidae** | 0 | 0 | 0 | 0 | 0 | 0 | 0 | * | 0 | 0 | 0 | 0 | 0 | * | 0 | * | * | 0 | 0 | 0 | 0 | 0 | * | 0 | * | 0 | 0 | * | 0 |
| **Simuliidae** | * | 1.3 | 8.3 | 0.1 | 0.2 | 44.3 | 0.2 | * | * | 0 | * | 14.4 | 0.3 | 40.6 | 0 | 1.1 | 61.2 | * | * | 43 | 0 | 0.1 | 13.4 | 7.1 | * | 22.8 | 0 | 0.1 | 5.1 |
| **Stratiomidae** | 0 | 0 | 0 | 0 | 0 | 0 | 0 | 0 | 0 | 0 | 0 | 0 | * | 0 | 0 | 0 | 0 | 0 | 0 | 0 | 0 | 0.5 | 0 | 0 | 0 | 0 | 0 | 0 | 0 |
| **Tabanidae** | 0 | 0 | 0 | 0 | 0 | 0 | 0 | 0 | 0 | 0 | 0 | 0 | 0 | 0 | 0 | 0 | 0 | 0 | 0 | * | 0 | 0 | 0 | 0 | 0 | 0 | 0 | 0 | * |
| **Tipulidae** | 0 | 0.1 | 0 | 0 | 0 | 0 | 0 | 0 | 0 | 0 | 0 | 0 | 0 | 0.2 | 0 | 0 | * | 0 | 0 | 0 | 0 | 2.8 | 0 | 0 | 0 | 0 | 0 | 0 | 0 |
| **Diptera pupae** | 0 | 0 | 0 | 0 | 0 | 0 | 0.1 | * | 0 | 4.3 | 0 | 0 | * | 0.4 | 0 | 0 | * | 0 | * | 0.2 | 0 | * | 0.1 | 0 | * | 0.2 | 0 | * | 0.2 |
| **Simuliidae pupae** | 0 | * | * | 0.9 | 0 | 2.8 | 0 | 0 | 0 | 0 | 0 | 0.6 | * | 0.6 | 0 | 0 | 1 | 0 | 0 | 2.5 | 0 | 0 | * | 0 | 0 | 3.2 | 0 | * | 0.2 |
| **Ephemeroptera** | 0 | 11.3 | 48.6 | 6.3 | 1.2 | 21.5 | 6.1 | 18.7 | 88.2 | 0 | 1 | 37.8 | 1.1 | 19.8 | 0 | 2.3 | 11.9 | 0 | 33.1 | 24.3 | 2.1 | 15.4 | 38.1 | 7.1 | 0.3 | 15.8 | 0 | 1.2 | 24.9 |
| **Trichoptera** | 0 | 1.4 | 19.5 | 4.9 | 0.3 | 20.8 | 15.5 | 12.1 | 0 | 0 | 0.4 | 39.9 | 7.3 | 15.7 | 0.1 | 2.1 | 17.4 | * | 0.7 | 3 | 1.9 | 2.8 | 11.9 | 3.6 | 0.4 | 33.3 | 9 | 3.4 | 22.7 |
| **Trichoptera pupae** | 0 | * | 0 | 0 | 0 | 0 | 0 | 0 | 0 | 0 | 0 | 0 | 0 | * | 0 | 0 | 0 | 0 | 0 | * | 0 | 0 | 0 | 0 | * | 0 | 0 | * | * |
| **Plecoptera** | 0 | 4.3 | 5.5 | 0 | 0.5 | 2.2 | 1.2 | 1.6 | 0 | 0 | 0.2 | 0.3 | 0.3 | 2.2 | 0 | 0.2 | 1.7 | 0 | 1 | 0.8 | 0 | 2.4 | 9 | 0 | * | 3.2 | 0 | * | 1.5 |
| **Odonata nymph** | 0 | 2.9 | 0 | 13.9 | 0.5 | 0 | 10.6 | 1.7 | 0 | 0 | 0 | 0 | 8.3 | 0.5 | 0 | 3.8 | 0 | 0 | 11.3 | 0 | 0 | 0.2 | 0 | 0 | 0 | 0 | 0 | 0.9 | 0 |
| **Coleoptera larvae** | * | 0.6 | 0 | 12.5 | 0.4 | * | 8 | 0 | 0 | 0 | 0 | 0 | 1 | 0 | 0 | 1.3 | * | 0 | 0 | 0 | 2.1 | 0.6 | 1.3 | 3.6 | * | 0 | 6 | 0.1 | 0.1 |
| **Aquatic Lepidoptera larvae** | 21.8 | 3.8 | 15.2 | 0.5 | 0.1 | 0 | 1.3 | 13.5 | 0 | 0 | 0.2 | 0.3 | 0 | 0 | 0 | 0.9 | * | 0 | 0.1 | 1.8 | 0.8 | 0.2 | 0 | 0 | 0 | 0 | 0 | 2.1 | 11.4 |
| **Terrestrial Lepidoptera larvae** | 0 | 0 | 0 | 0.2 | 0 | 0 | 4.8 | 0 | 0 | 0 | 0 | 0 | 0 | 0 | 0 | 0 | 0 | 0.8 | 0 | 0 | 0 | 0.8 | 0 | 0 | 70.5 | 0 | 0 | 0 | 0 |
| **Aquatic Hemiptera** | 0 | 0.2 | 0 | 0 | 0 | 0 | 0 | 0 | 0 | 0 | 0 | 0 | 0 | 0 | 0 | 0 | 0 | 0 | 0 | 0 | 0 | 0 | 0 | 0 | 0 | 0 | 0 | 0.2 | 0 |
| **Aquatic insect remains** | 0 | 0.6 | 0.4 | * | 0.3 | 0.6 | 0.9 | 23.8 | 0 | 0 | 0.4 | * | 5.3 | 1.7 | 0.4 | 0.2 | 0.2 | 0 | 0 | 0.4 | 2.5 | 2.1 | 1.8 | 3.6 | 0.3 | 0 | 0 | 0.4 | 0.6 |
| **Adult Coleoptera** | 0 | 0.3 | 0 | 6.9 | * | 0 | 4.4 | 0.2 | 0 | 0 | * | 0 | 7.7 | 0 | 0 | 3 | * | 0 | 0 | 0 | 8.4 | * | 0 | 3.6 | 0 | 0 | 6 | 0.2 | 0 |
| **Adult Diptera** | 0 | 0 | 0 | 0.5 | 0 | 0 | 0.1 | 0 | 0 | 0 | 0 | 0 | 0 | 0 | 0 | 0 | 0.2 | 0 | 0 | 0 | 0 | 0 | 0 | 0 | * | 0 | 0 | * | 0 |
| **Adult Lepidoptera** | 0 | 0.1 | 0.2 | 0 | 0 | 0 | 0 | 0 | 0 | 0 | 0 | 0 | 0 | 0 | 0 | 0 | 0 | 0 | 0 | 0 | 0 | 0 | 0 | 0 | 0 | 0 | 0 | 0.2 | 0 |
| **Hymenoptera** | 0.4 | * | 0 | 1.3 | 0.5 | 0 | 0 | 0 | 0 | 0 | 0 | 0 | 0.1 | 0 | 0 | 0 | 0 | 0 | 0 | 0 | 10.6 | 0 | 0 | 7.1 | 0.3 | 0 | 0 | 0.6 | 0.4 |
| **Adult Odonata** | 0 | 0 | 0 | 0 | 0 | 0 | 0 | 4.5 | 0 | 0 | 0 | 0 | 0 | 0 | 0 | 0 | 0 | 0 | 0 | 0 | 0 | 4 | 0 | 0 | 0 | 0 | 0 | 0.4 | 0 |
| **Homoptera** | 0 | 0 | 0 | 0 | 0.5 | 0 | 0 | 0 | 0 | 0 | 0 | 0 | 0 | 0 | 0 | 0 | 0 | 0 | 0 | 0 | 0 | 2 | 0 | 0 | 0 | 0 | 0 | 0 | 0 |
| **Mantodea** | 0 | 0 | 0 | 0.1 | 0 | 0 | 0 | 0 | 0 | 0 | 0 | 0 | 0 | 0 | 0 | 0 | 0 | 0 | 0 | 0 | 0 | 0 | 0 | 0 | 0 | 0 | 0 | 0 | 0 |
| **Terrestrial Hemiptera** | 0 | 0 | 0 | 0 | 0 | 0 | 0 | 0 | 0 | 0 | 0 | 0 | 0 | 0 | 0 | 0 | 0 | 0 | 0 | 0 | 0 | 0 | 0 | 0 | 0 | 0 | 1.6 | * | 0 |
| **Terrestrial insect remains** | 0.5 | 1 | 0 | 5.1 | 0.1 | 0.1 | 6.9 | 0.6 | 0 | 34.8 | 0.4 | 0 | 1.4 | 2.7 | 0 | 0.8 | 0 | 0.3 | 0 | 1.8 | 20.9 | 0 | 0 | 10.7 | * | 0 | 2.4 | 0.7 | 0.3 |
| ***Aegla* sp.** | 0 | 0.2 | 0 | 16.1 | 67.9 | 0 | 2.9 | 0 | 0 | 0 | 54.5 | 0 | 21.5 | 0 | 0.6 | 45 | 0 | 26.6 | 41.1 | 0.7 | 21.1 | 46 | 0 | 7.1 | 23.6 | 0 | 13.9 | 58.2 | 0 |
| **Araneae** | 0 | 0 | 0 | 7.3 | 0 | 0.1 | 0.4 | 0 | 0 | 0 | 0 | 0 | 0.5 | 0 | 0 | 0 | 0 | 0 | 0 | 0 | 1.3 | 0 | 0 | 0 | 0 | 0 | 0 | * | 0 |
| **Diplopoda** | 0 | 0 | 0 | 0 | 0 | 0 | 9.6 | 0 | 0 | 0 | 0 | 0 | 0 | 0 | 0 | 0 | 0 | 0 | 0 | 0 | 0 | 0 | 0 | 3.6 | 0.5 | 0 | 1.8 | 0 | 0 |
| **Isopoda** | 0 | 0 | 0 | 0 | 0 | 0 | 0 | 0 | 0 | 0 | 0 | 0 | 0.3 | 0 | 0 | 0 | 0 | 0 | 0 | 0 | 0 | 0 | 0 | 0 | 0 | 0 | 0 | 0 | 0 |
| **Megaloptera** | 0 | 8.3 | 0 | 0 | 21.4 | 0 | 0 | 0 | 0 | 0 | 39.3 | 0.4 | 3.2 | 0.3 | 0 | 36.4 | 1.7 | 0 | 0 | 0 | 0 | 0 | 0 | 0 | 0 | 0 | 0 | 18.5 | 0 |
| **Acarina** | 0 | * | 0 | 0 | 0 | 0 | * | * | 0 | 0 | 0 | 0 | 0 | 0 | 0 | 0 | 0 | 0 | 0 | 0 | 0 | 0 | 0 | 0 | * | 0 | 0 | 0 | * |
| **Cladocera** | 0 | 0 | 0 | 0 | 0 | * | 0 | 0 | 0 | 0 | 0 | 0 | 0 | 0 | 0 | 0 | * | 0 | 0 | * | 0 | 0 | 0 | 0 | 0 | 0 | 0 | * | * |
| **Conchostraca** | 0 | 0 | 0 | 0 | * | 0 | 0 | 0 | 0 | 0 | 0 | 0 | 0 | 0 | 0 | 0 | 0 | 0 | 0 | 0 | 0 | 0 | 0 | 0 | 0 | 0 | 0 | 0 | 0 |
| **Copepoda** | 0 | 0 | * | * | * | 0 | 0 | 0 | 0 | 0 | 0 | 0 | 0 | 0 | 0 | 0 | 0 | 0 | * | 0 | 0 | * | * | 0 | * | 0 | 0 | * | 0 |
| **Ostracoda** | 0 | 0 | 0 | 0 | 0 | * | * | * | 0 | 0 | 0 | 0 | 0 | 0 | 0 | 0 | * | 0 | 0 | 0 | 0 | 0 | 0 | 0 | 0 | 0 | 0 | 0 | 0 |
| **Bivalve** | 0.8 | * | 0 | 0.2 | 0.5 | 0 | 0 | * | 0 | 0 | 0 | 0 | 0 | 0 | 0 | 0 | 0 | 0 | 0 | 0 | 0.1 | 0 | 0 | 0 | 0 | 0 | 0 | 0 | 0 |
| **Gastropoda** | 0 | * | 0 | 1.6 | 0 | 0 | 3.9 | 2.4 | 0 | 0 | 0 | 0 | 0 | 0 | 0 | 0 | 0 | 0 | 0 | 0 | 2.5 | 0 | 0 | 3.6 | 0 | 0 | 0 | 0 | 0 |
| **Unidentified Mollusca** | 0 | * | 0 | 0 | 0 | 0 | 0 | 0.2 | 0 | 0 | 0 | 0 | 0 | 0 | 0 | 0 | 0 | 0 | 0 | 0 | 0 | 0 | 0 | 0 | 0 | 0 | 0 | 0 | 0 |
| **Oligochaeta** | 0 | 0.6 | 0 | 13.6 | 0 | 0 | 15.3 | 0.9 | 0 | 0 | 0 | 1 | 27.3 | 0 | 97 | 0.7 | 0.6 | 2.9 | 0 | 0.2 | 0 | 11.5 | 1.1 | 7.1 | 1 | 1.3 | 0 | 3.2 | 3.2 |
| **Nematoide** | 0 | * | * | 0 | 0 | 0.1 | 1.3 | 0 | 0 | 0 | * | 0.1 | 0 | * | 0 | 0 | * | 0 | * | 0.2 | 0 | 0 | * | 3.6 | * | * | 15 | * | 0.1 |
| **Platyelmintes** | 0 | 0 | 0 | 0 | 0 | 0.2 | 0 | 0 | 0 | 0 | 0 | 0 | 0 | 0 | 0 | 0 | 0 | 0 | 0 | 0 | 0 | 0 | 0 | 0 | 0 | 0 | 0 | 0 | 0 |
| **Testate Amoebae** | 0 | * | 0 | 1 | * | * | 0.1 | * | 0 | 0 | 0 | 0 | 0 | 0 | 0 | 0 | 0 | 0 | 0 | 0 | * | 0 | 0 | 0 | 0 | 0 | 0 | 0 | 0 |
| **Scale** | * | * | * | * | 0.2 | 0 | 1.3 | * | 0 | 0 | 0 | 0 | * | 0 | 0 | 0 | * | 0 | 0 | * | 10.2 | * | * | 3.6 | * | * | 6 | * | * |
| **Fish** | 0 | 11.8 | 0 | 0 | 4 | 0 | 0 | 0.6 | 0 | 0 | 2.9 | 0 | 0 | 0 | 0 | 0.9 | 0 | 68.7 | 0 | 0 | 0 | 3.2 | 0 | 10.7 | 1 | 0 | 16.2 | 2 | 0.4 |
| **Fur** | 0 | 0 | 0 | 0 | 0 | 0 | 0 | 0 | 0 | 0 | 0 | 0 | 0 | 0 | 0 | 0 | 0 | 0 | 0 | 0 | 0 | 0 | 0 | 0 | 0 | * | 0 | 0 | 0 |
| **Aquatic plant remains** | 0 | 0.2 | 0 | 0 | 0 | 0 | 0 | 0.2 | 0 | 0 | 0 | 0 | 0.1 | 0 | 0 | 0 | 0 | 0 | 0 | * | 0 | 0 | 0 | 0 | 0 | 0 | * | 0.1 | 0 |
| **Terrestrial plant remains** | 76.2 | 49.3 | 0.3 | 6.3 | 0.7 | 0.3 | 3.8 | 1.5 | 0 | 60.9 | 0.4 | 0.6 | 11.1 | 1.2 | 0.1 | 0.6 | 0.4 | 0.7 | 11.2 | 1.3 | 5.7 | 1.3 | 2.6 | 7.1 | 0.6 | 4.3 | 10.1 | 5.4 | 8.7 |
| **Animal organic matter** | 0 | 0.2 | 0 | 0.4 | 0.1 | 0.4 | * | 10.4 | 0 | 0 | 0 | 0 | 1.6 | 1.4 | 1.8 | 0 | * | 0 | 0 | 14.1 | 7.6 | 0 | 0 | 0 | 0.7 | 0.6 | 0 | * | 0.2 |
| **Vegetal organic matter** | 0 | 0 | 0 | 0 | 0 | 0 | 0 | * | 0 | 0 | 0 | 0 | 0.6 | 0 | 0 | * | 0 | 0 | 0 | 0 | 0 | 0 | 0 | 0 | 0 | 0 | 0 | * | 0 |
| **Undetermined organic matter** | 0 | 0 | 0.4 | 0 | 0 | 0.2 | 0.2 | 1 | 0 | 0 | 0 | 0 | 0 | * | 0 | 0 | 0 | 0 | 0 | 0 | 0 | 0 | 0 | 0 | 0 | 0.5 | 0 | * | 1.1 |
| **Detritus** | 0 | 0 | 0 | 0 | 0 | 0 | 0 | 0 | 0 | 0 | 0 | 0.1 | 0.6 | 0 | 0 | 0.5 | 0 | 0 | * | 0 | 0 | 0.1 | 0 | 0 | * | 0 | 0.1 | 0 | 0 |
| **Sediment** | 0 | 1.1 | * | 0 | 0.3 | 0.2 | 0.8 | 5.2 | 8.7 | 0 | 0.1 | 0.4 | 0.2 | 0.9 | 0 | 0.1 | * | 0 | 0.3 | * | 0.2 | 2.6 | 0.3 | 3.6 | 0.4 | 0.1 | 0 | 0.9 | 5.1 |
